# Connected Embedded System for Drowsiness Detection Based on a Reconfigurable Set of Features

**DOI:** 10.3390/s26041195

**Published:** 2026-02-12

**Authors:** Ibtissam Belakhdhar

**Affiliations:** IRSEEM (UR 4353), ESIGELEC, Universite de Rouen Normandie, 76800 Saint-Etienne-du-Rouvray, France; ibtissam.belakhdhar@irseem-esigelec.fr

**Keywords:** EEG, ANN, IOT, FFT

## Abstract

In this study, we present a new EEG-based drowsiness-detection system using a single EEG channel and IoT technology. The aim of this work is to develop a person-dependent system capable of overcoming interpersonal variability due to aging while sending alert signals to the cloud. We used a set of five features computed from the power spectral density, based on variations in power spectral energy during the transition from wakefulness to drowsiness (stage one of sleep) for each individual. The results demonstrate that the proposed system can accurately detect driver drowsiness, achieving an accuracy of 95% using a reduced set of features and a single differential EEG channel. The main advantage of the proposed system lies in its ability to overcome interpersonal variability while maintaining high detection accuracy. The system was validated using the MIT-BIH Polysomnography dataset, comprising ten subjects.

## 1. Introduction

Between 1980 and 2000, various studies focused on using behavioral indicators to develop drowsiness-detection systems. These systems monitored different tasks performed by the driver, such as steering wheel angle and braking, to determine a driver’s state. Although these systems yielded satisfactory results, they were difficult to implement due to the specialized and expensive equipment required. Other studies used driver face analysis [1,2,3] to determine a driver’s condition. This approach is noninvasive, easy to implement, and works with any type of car. However, the accuracy of systems based on this approach varies and can be affected by lighting conditions and the distance between the driver and the camera. Some studies [2] reported an accuracy of 98.2%, but it is important to note that the performance of these systems is not consistent in different environments. Moreover, there are other methods that used biomedical, electrocardiographic, and electrooculographic signals [4] to detect driver drowsiness. These signals are collected from the body during the sleep cycle. However, the most-used one is the electroencephalography (EEG), which it is considered as a standard technique in sleep studies. On the other hand, the systems based on biomedical signals require the attachment of sensors and wires to the body. This problem can be solved by using a reduced number of channels and wireless sensors for the acquisition of signals. The EEG measures the electrical activity of the brain using electrodes placed on the scalp. In fact, it is a set of rhythmic activities, represented by frequency bands, which are the delta (δ) activity [0:5–4] Hz, theta (θ) activity [4–8] Hz, alpha (α) activity [8–12] Hz, beta (β) activity [12–26] Hz, and gamma(γ) activity (over 26 Hz) bands. Only the range of [1–20] Hz is considered useful when detecting drowsiness, whereas the rest of the frequencies are considered as noise.

In this work, we propose an embedded and connected system for EEG-based drowsiness detection built around an ARM processor. The proposed system detects drowsiness using a single differential EEG channel and transmits alert signals to the ThingSpeak cloud platform via the Wi-Fi module integrated into a Raspberry Pi device, making it suitable for real-time and low-complexity applications. Most existing drowsiness-detection approaches rely on fixed EEG frequency bands defined in a conventional manner. However, neuroscience studies have shown that the boundaries of these bands vary significantly across individuals, particularly as a function of age, which limits the generalization performance of subject-independent systems. Motivated by these findings, we introduce a neuroscience-inspired and reconfigurable set of five EEG features that adapts to individual spectral characteristics [5,6,7]. This design enables the development of a subject-dependent drowsiness-detection system capable of reducing the effects of interpersonal variability while preserving high detection accuracy. Furthermore, the proposed approach is specifically designed for embedded implementation, combining low computational complexity with reduced hardware requirements. By relying on a single EEG channel and a compact feature set, the system achieves accurate drowsiness detection while improving usability and comfort compared to multi-channel EEG-based solutions. The remainder of this paper is organized as follows. Section 2 reviews related work. Section 3 presents the proposed algorithm. Section 4 describes the embedded system architecture. Section 5 discusses the experimental results, and Section 6 concludes the paper.

## 2. Related Work

Drowsiness is a major contributing factor in road accidents, as it reduces a driver’s ability to concentrate and make quick decisions. Several automatic drowsiness-detection approaches based on electroencephalogram (EEG) signals have been proposed in the literature. However, only a limited number of these studies focused on embedded systems for real-time drowsiness detection. In this section, we review relevant work, specifying the embedded processing units used and the accuracy achieved.

Chinara et al. [8] developed a drowsiness-detection system using temporal sub-bands extracted from EEG signals via Wavelet Packet Transform (WPT). Feature selection was performed using the Mann–Whitney U Test (MWUT), producing a vector of four features classified with the Extra Tree algorithm. The system achieved an accuracy of 85.4%.

Bajaj et al. [9] proposed a system using five features extracted from the time domain through the Tunable Q-factor Wavelet Transform (TQWT) method. Classification was performed using an Extreme Learning Machine (ELM), reaching an accuracy of 91.84%.

Wang et al. [10] validated their system using the Expanded (EDF) database. Feature vectors were extracted from EEG signals using db10 and Haar Wavelet Packet Transforms and classified with a Support Vector Machine (SVM), achieving an accuracy of 89.52%.

Budak et al. [11] combined temporal and frequency representations of EEG signals. Statistical features were extracted using AlexNet, VGGNet, and TQWT, and then fed into a Long Short-Term Memory (LSTM) network. Experimental results on the MIT-Polysomnography (Massachusetts Institute of Technology Polysomnography) dataset demonstrated an accuracy of 94.31%.

Tripathy et al. [12] implemented an embedded system composed of wireless EEG acquisition and processing on an Analog Devices Signal Processor Blackfin BF533 (ADSP-BF533) processor running on u-Clinux. Signals from three EEG electrodes were filtered, transformed using Fast Fourier Transform (FFT), and classified via Mahalanobis distances for theta (MDT) and alpha (MDA) rhythms. This system achieved 88.4% accuracy.

Lin et al. [13] proposed a four-part embedded system with acquisition/amplification, wireless transmission, dual-core processing (Advanced RISC Machine (ARM) + Digital Signal Processor (DSP)), and a host system. It detected driver drowsiness with an average accuracy of 74.6%.

Jiang et al. [14] developed a mobile and wireless EEG system to monitor driver vigilance and relate brain activity changes to driving performance. In [15], an automatic EEG-based drowsiness detection method was proposed using spectral, wavelet, and time domain features. Seven features were extracted from a single EEG channel to discriminate between alert and drowsy states. Likewise, ref. [16] also relied on a single EEG channel, where feature vectors were computed using Power Spectral Density (PSD), Wavelet Transform (WT), and the Burg method, and classified using neural networks. Despite their effectiveness, these approaches involve computationally intensive feature-extraction processes, which limits their suitability for real-time embedded implementations. Most existing EEG-based drowsiness-detection systems use a single model for all subjects. However, inter-subject variability, including age-related differences in EEG bands [17], requires personalized models. Regression-based methods are commonly used, but they cannot fully adapt to instantaneous variations.

In contrast, our approach develops an embedded system that is easily integrable in vehicles and adapts to individual variations. We use a configurable set of neuroscience-inspired features [17], achieving a high accuracy of 96%, demonstrating its effectiveness in overcoming inter-subject variability.

## 3. Proposed Architecture for Drowsiness-Detection System

### 3.1. Proposed Method

**Figure 1:**
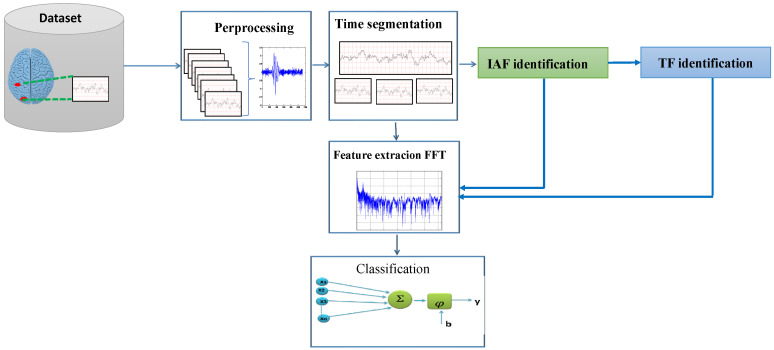
Flowchart of the drowsiness-detection approach.

In this article, we introduce a novel drowsiness-detection system based on IOT and EEG signal. This system utilize a set of four new features. Figure 1 presents the flow chart of our proposed system.

In this work, the MIT-BIH Polysomnography dataset, which is available online, is used [18]. This dataset contains the recordings of 16 persons with different physiologic signals during sleep. The aim of this work is to detect the appearance of micro-sleep; therefore, only the records relating to the awake state (W) and stage I of sleep (S1) are extracted from the database [18]. Next, the signals are filtered by the Butterworth filter of the second order with cut of frequencies of [0.5–50] Hz. Then, to guarantee the statistical stationarity necessary for the calculation of power spectrum density, the signal divided into segments of 30 s each.

#### 3.1.1. Feature Extraction

The calculation of this new configurable set of features is carried out in two stages: in the first stage, the Individual Alpha Frequency (IAF) of each person is calculated. Then, after the identification of the IAF for each person, the TF frequency for each person is calculated, which is called the Theta Frequency [17] which is IAF frequency −4. Using these two frequencies and based on the variation of power frequency during the awake–sleep stage I transition, we have calculated five features. In fact, the largest increase in EEG power occurs at 3 and 4 Hz [17,19], whereas the greatest decrease in EEG power occurs at 9, 10, and 11 Hz. Based on these results, we extracted five features from the PSD of the EEG for each epoch: (1) the power at the Individual Alpha Frequency (IAF), (2) the power at the theta frequency (TF), (3) the power at 3 Hz, (4) the power at 4 Hz, and (5) the ratio of the average power in the 3–4 Hz band to the average power in the alpha band (8–12 Hz).

#### 3.1.2. Characteristic Vector Calculation Procedure

**Figure 2:**
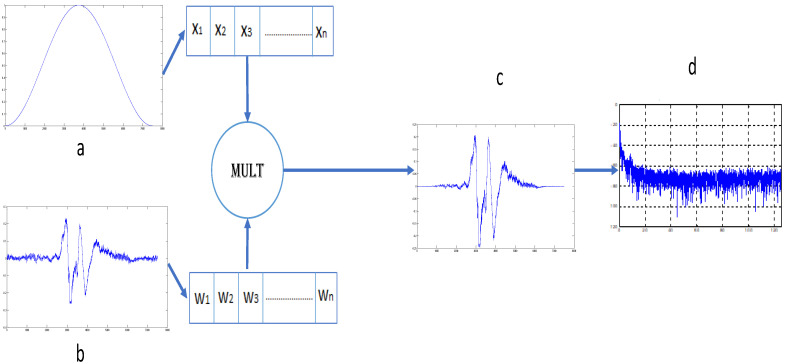
(**a**) Hanning window N, (**b**) epoch of size of size N, (**c**) multiplication result and (**d**) FFT.

Firstly, for each epoch corresponding to the awake (W) or stage1(S1), the FFT [7] was calculated using a Hanning window, as shown in Figure 2: firstly, the signal was multiplied by the Hanning window according to Equation (1).

(1) $$w\left(n\right)=\frac{1}{2}\left(1-\cos\left(\frac{2\pi n}{N-1}\right)\right)$$

where w(n) is the window function applied to a sequence of discrete data, *n* is the index of the current data, *N* is the total number of data in the sequence.

The FFT of each segment of 30 s of the EEG signal

Y(n)=y(1),y(2),…,y(N)

is given by Equation (2)

(2) $$X_{K}=\sum_{n=0}^{N-1}Y\left(n\right)e^{-i2\pi kn/N}\;k=0,1,2,\ldots ,N-1\;$$

The power spectral density is defined as:

(3) $$PSD\left(k\right)=\frac{1}{N}\;{\left|X\left(k\right)\right|}^{2}$$

After calculating the power spectral density (PSD), the Individual Alpha Frequency (IAF) is first determined. The IAF corresponds to the frequency with the maximum power within the alpha band, defined as 8–12 Hz. The theta frequency (TF) is then defined as:

(4) TF=IAF−4

After determining these two frequencies, the power at the (IAF), (TF), 3 Hz, and 4 Hz was calculated using Equation (5).

(5) $$P\left(f\right)=PSD\left(k_{f}\right)$$

After calculating these four features, the fifth feature was computed using Equation (6) as the ratio between the average power in the 3–4 Hz band and the average power in the alpha band.

(6) $$R=\frac{\frac{1}{N_{3-4}}\sum_{k=k_{3}}^{k_{4}}PSD\left(k\right)}{\frac{1}{N_{\alpha }}\sum_{k=k_{\alpha }^{\left(1\right)}}^{k_{\alpha }^{\left(2\right)}}PSD\left(k\right)}$$

#### 3.1.3. Classification

When developing an algorithm for drowsiness detection using EEG, both regression and classification techniques can be used. However, classification techniques are more commonly employed, with algorithms such as SVM [20,21], LDA [22], and ANN [23] being popular choices. Among these options, ANN is the most frequently utilized [5], with the multilayer perceptron (MLP) being the most commonly implemented type of ANN. The MLP consists of multiple layers, including an input layer, a hidden layer (with a number of neurons determined empirically), and an output layer that identifies the class of the output vector.

### 3.2. The Proposed Embedded Implementation of a Connected Drowsiness Monitoring System for Vehicles

We chose to use a Raspberry Pi development board for our drowsiness-detection system for several reasons. Firstly, the board comes with integrated wireless communication protocols, such as WiFi and Bluetooth, which make it easy to communicate with the cloud and EEG helmets (such as EPOC, Emotiv, etc.) via Bluetooth. Secondly, the board is compatible with the pi-EEG device, which simplifies the acquisition and processing of EEG signals. Thirdly, the device is compatible with Carberry Pi, which facilitates communication between the Raspberry Pi and car electronics.

## 4. Experimentation and Results

The aim of this section is to conduct a scientific experiment and demonstrate the effectiveness of using a multilayer neural network with wearable EEG for detecting drowsiness in vehicle drivers.

### 4.1. Dataset

To validate our drowsiness-detection system, we used a sleep-related database, as drowsiness corresponds physiologically to the transition between wakefulness and stage 1 sleep, and such data are commonly used in studies on driver vigilance [15]. Among several available databases, including MIT-BIH Polysomnography [18], CAP Sleep EEG [24], and the extended Sleep-EDF [25], we selected MIT-BIH Polysomnography for its sufficient number of well-annotated recordings of wakefulness and light sleep. Sleep stages were scored by experts on 30 s epochs according to Rechtschaffen and Kales [26], and only epochs labeled “W” (wakefulness) and “S1” (stage 1) were used, corresponding to alert and drowsy states, respectively. The database contains recordings from 16 subjects with multiple physiological signals, including EEG recorded via two differential channels (A1–C4 and C3–O1) at 250 Hz. To reduce system complexity and improve user comfort, only the C3–O1 channel was considered, and only the EEG signals from the ten subjects with available C3–O1 recordings (p03, p04, p14, p16, p48, p59, p60, p61, p66, and p67) were used in this study.

### 4.2. Evaluation Metrics

We evaluated the performance of the classifier using three evaluation metrics derived from the confusion matrix: the True Positive Rate (TPR), the True Negative Rate (TNR), and the overall accuracy. The confusion matrix is a table that summarizes the performance of a classification model by comparing the predicted labels with the true labels, allowing us to see how many predictions were correct and how many were misclassified. The evaluation metrics are defined as follows:

(7) $$\mathrm{True}\;\mathrm{Positive}\;\mathrm{Rate}\;\left(\mathrm{TPR}\right)=\frac{\mathrm{TP}}{\mathrm{TP}+\mathrm{FN}}$$

(8) $$\mathrm{True}\;\mathrm{Negative}\;\mathrm{Rate}\;\left(\mathrm{TNR}\right)=\frac{\mathrm{TN}}{\mathrm{TN}+\mathrm{FP}}$$

(9) $$\mathrm{Accuracy}=\frac{\mathrm{TP}+\mathrm{TN}}{\mathrm{TP}+\mathrm{TN}+\mathrm{FP}+\mathrm{FN}}$$

where: True Positive (TP): The classifier correctly predicts sleepiness when the subject is actually in a sleepy state. False Negative (FN): The classifier incorrectly predicts alertness when the subject is actually sleepy. True Negative (TN): The classifier correctly predicts alertness when the subject is actually alert. False Positive (FP): The classifier incorrectly predicts sleepiness when the subject is actually alert. The True Positive Rate (TPR), also called sensitivity or recall, measures the classifier’s ability to correctly detect sleepy states. The True Negative Rate (TNR), also called specificity, evaluates its ability to correctly recognize alert states. Finally, the overall accuracy represents the proportion of correctly classified instances among all predictions.

### 4.3. Implementation Procedure and Results

**Figure 3:**
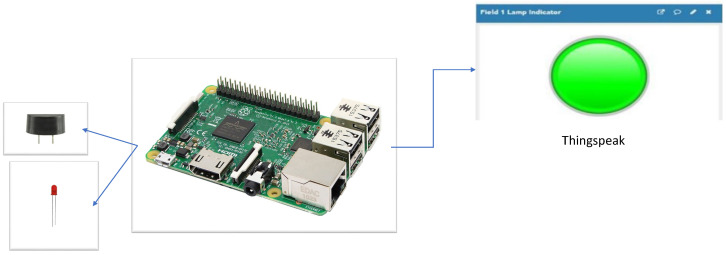
The proposed architecture of a connected drowsiness monitoring system for vehicles.

The proposed system architecture is illustrated in Figure 3. The system was implemented using the C programming language on a Raspberry Pi platform. When the system detects that the driver is drowsy, it generates a local alert using a buzzer. Simultaneously, an alert signal is transmitted to the cloud to notify the supervisor that the truck driver is in a drowsy state and should stop driving in order to prevent potential accidents. ThingSpeak was selected as the cloud platform, as it enables real-time monitoring of the driver’s vigilance status. Specifically, a red LED is activated when the driver is drowsy, whereas a green LED indicates an alert state.

To implement the proposed approach on the Raspberry Pi, two software libraries were used: FFTW3, employed for spectral analysis and computation of the power spectral density (PSD), and FANN (Fast Artificial Neural Network), used for implementing the MLP classifier.

Feature extraction was carried out as follows. First, the acquired signal was filtered using a Butterworth filter and then segmented into 30 s epochs. For each segment labeled as drowsy (S1) or awake, a Fast Fourier Transform (FFT) was applied using a Hanning window corresponding to the number of samples per segment, followed by the computation of the PSD. After estimating the PSD for each 30 s segment, the Individual Alpha Frequency (IAF) was first extracted from the power spectrum, and the TF frequency was subsequently derived from the IAF. Based on these two frequencies, the complete feature set was extracted for each subject.

The classification stage was performed using a three-layer Multilayer Perceptron (MLP) neural network. The network consists of an input layer with five neurons, corresponding to the extracted features, a hidden layer, and an output layer with a single neuron that determines the subject’s vigilance state (alert or drowsy) using a tangent sigmoid activation function. For each subject, the number of neurons in the hidden layer was empirically optimized during the training phase by varying it from 5 to 25 neurons in steps of five (5, 10, 15, 20, and 25). It was observed that no further improvement in classification accuracy was achieved beyond 25 neurons.

To ensure the reliability and robustness of the proposed classification framework, a 10-fold cross-validation scheme was employed. The feature vectors of each subject were divided into 10 folds, where 9 folds were used for training and 1 fold was used for testing. This procedure was repeated 10 times, ensuring that each fold was used exactly once as the test set. The results of the testing session are summarized in Table 1. The TPR for drowsiness ranged from 80 % to 100%, with an average of 94.29%, while the TNR averaged 95.87%, reaching 100% at its maximum. These high-performance values indicate that the proposed system can reliably detect drowsiness and support driver safety.

## 5. Discussion

In this paper, we propose a person-dependent drowsiness-detection system using a single differential EEG channel. By relying on only one channel, our approach ensures high detection accuracy while remaining user-friendly and comfortable for the driver. Table 2 provides a comparative overview of existing methods and the proposed system, highlighting differences in dataset, number of electrodes, preprocessing, feature extraction, number of features, classification method, and achieved drowsiness detection accuracy. As shown, the proposed system outperforms previous approaches, achieving the highest accuracy of 95.07% while using a minimal number of electrodes, demonstrating its efficiency and suitability for embedded implementation.

The approach proposed for drowsiness detection in [11] is relevant and it achieves a high accuracy of 94.31% in detecting driver drowsiness, which proves that this method is very effective. However, compared to our approach, it is very complex. In fact, for feature extraction, it uses three methods, and during classification, it uses a LSTM classifier and the method of majority vote to classify a person’s state accurately.

Researchers in [10] developed a drowsiness-detection system that utilizes information from the frequency band [43.75–48.046875] Hz and can detect drowsiness with an accuracy of 89.52%. However, this system is less comfortable to use than our approach since it requires signals to be recorded from four electrodes, whereas our system only needs two electrodes despite achieving similar accuracy.

Researchers in [8] have developed a drowsiness-detection system that achieves similar accuracy to our approach, but their method employs more complex techniques than ours. Specifically, their system utilizes the Permutation Entropy-Weighted Transform (PWT), which is more complex than our use of Fast Fourier Transform (FFT) for feature extraction.

Although the system proposed by [9] uses a reduced number of electrodes, its accuracy in detecting drowsiness is only 91.84%, which is lower than that achieved by our system. In contrast, the system presented in [27] employs more features than our approach but still achieves a similar level of accuracy of 88.80%.

In [7], the authors proposed a drowsiness-detection system using the same technique employed in our work. However, our proposed system introduces a more compact and new set of features, resulting in a 9% improvement in decision accuracy compared to their system.

## 6. Conclusions

In this paper, we present an embedded system for drowsiness detection. Specifically, we have implemented a person-dependent algorithm on an ARM processor. The developed system can send an alert signal to the cloud using wireless protocol. To validate the performance of the proposed system, we used the MIT-BIH Polysomnography public dataset, and achieved a precision of 94% in detecting drowsiness. Thanks to its high precision and compact size, the developed system can be easily integrated into a car. Therefore, it has the potential to reduce the rate of accidents caused by drowsiness by detecting the state of the driver and alerting them if they are becoming drowsy. This application can be deployed by road transport companies to assist drivers making long journeys, who are often exposed to the risks associated with drowsiness at the wheel.

## Figures and Tables

**Table 1:** Test result for each person using Matlab.

	Age	TPN	TNR	Accuracy
p03	51	80	100	90
p04	35	100	100	100
p14	40	100	95	97.5
p16	35	88.9	85.7	87.3
p48	56	100	100	100
p59	41	100	100	100
p60	49	83	88	85.5
p61	32	100	100	100
p66	33	90.9	90	90.45
p67	–	100	100	100
Average	35	94.29	95.87	95.07

**Table 2:** Comparison of drowsiness-detection systems.

	[11]	[10]	[8]	[9]	[27]	[7]	Proposed System
Dataset	MIT-BIH Polysomnography	Sleep_EDF [expanded]				MIT-BIH Polysomnography	MIT-BIH Polysomnography
Number of electrodes	1		1			1	
Preprocessing				Butter worth		Butter worth	
Feature extraction	TQWT/Alexnet/VGGN	Wavelet packet transform	WPT	TQWT	FFT	FFT	FFT
Number of features	4	2	66			5	4
Classification	LSTM	SVM	Extra Trees	ELM	LSTM	MLP	MLP
Accuracy of drowsiness detection	94.31%	89.52 %	94.45%	91.84%	88.80%	84.4%	95.07

## Data Availability

The data analyzed in this study are publicly available in the PhysioNet repository, specifically the Sleep-EDF Expanded Database (SLPDB), at https://archive.physionet.org/physiobank/database/slpdb/ (accessed on 3 February 2026).
